# Doxorubicin-loaded Cu_2_S/Tween-20 nanocomposites for light-triggered tumor photothermal therapy and chemotherapy†

**DOI:** 10.1039/d0ra03069d

**Published:** 2020-07-10

**Authors:** Shuwei Liu, Lu Wang, Bin Zhao, Ze Wang, Yinyu Wang, Bin Sun, Yi Liu

**Affiliations:** Department of Oral and Maxillofacial Surgery, School and Hospital of Stomatology, Jilin University Changchun 130021 P. R. China sunbin06@sohu.com; Jilin Provincial Key Laboratory of Tooth Development and Bone Remodeling, Jilin University Changchun 130021 P. R. China; State Key Laboratory of Supramolecular Structure and Materials, College of Chemistry, Jilin University Changchun 130012 P. R. China; School of Stomatology, Baicheng Medical College Baicheng 137000 P. R. China

## Abstract

In clinical tumor therapy, traditional treatments such as surgery, radiotherapy and chemotherapy all have their own limitations. With the development of nanotechnology, new therapeutic methods based on nanomaterials such as photothermal therapy (PTT) have also emerged. PTT takes advantage of the poor thermal tolerance of tumor cells and uses the heat generated by photothermal reagents to kill tumor cells. A transition metal sulfide represented as Cu_2_S is an ideal photothermal reagent because of its easy preparation, high extinction coefficient and photothermal conversion efficiency. Surface modification of nanoparticles (NPs) is also necessary, which not only can reduce toxicity and improve colloidal stability, but also can provide the possibility of further chemotherapeutic drug loading. In this work, we report the fabrication of Tween-20 (Tw20)-modified and doxorubicin (Dox)-loaded Cu_2_S NPs (Cu_2_S/Dox@Tw20 NPs), which significantly improves the performance in tumor therapy. Apart from the enhancement of colloidal stability and biocompatibility, the drug loading rate of Dox in Tw20 reaches 11.3%. Because of the loading of Dox, Cu_2_S/Dox@Tw20 NPs exhibit chemotherapeutic behaviors and the tumor inhibition rate is 76.2%. Further combined with a near-infrared laser, the high temperature directly leads to the apoptosis of a large number of tumor cells, while the release of chemotherapeutic drugs under heat can not only continue to kill residual tumor cells, but also inhibit tumor recurrence. Therefore, with the combination of PTT and chemotherapy, the tumor was completely eliminated. Both hematological analysis and histopathological analysis proved that our experiments are safe.

## Abstract 2

In clinical tumor therapy, traditional treatments such as surgery, radiotherapy and chemotherapy all have their own limitations.

## Graphical abstract

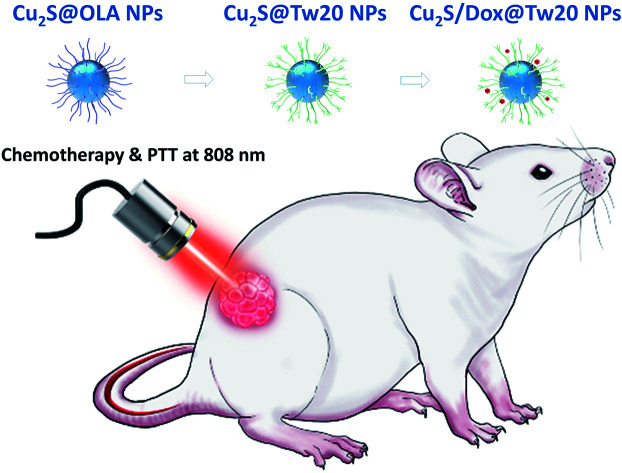

## 1 Introduction

For decades, cancer has been the biggest threat to human health.^1^ New cases and deaths due to cancer are rising every year.^2^ Traditional treatments all have their own limitations.^3^ For example, surgical treatment is operator dependent and recurrence due to incomplete resection is very common.^4^ In surgical treatment, patients often suffer great pain.^5^ Moreover, radiotherapy and chemotherapy will cause high toxic and side effects.^6^ Therefore, for more than a decade, with the quick development of nanotechnology, new therapeutic methods including mainly phototherapy,^7,8^ gene therapy,^9,10^ and immune-therapy^11–13^ based on nanomaterials have also emerged.

Among them, the research on phototherapy is very extensive, and there are many kinds of phototherapy reagents, which have a wider application.^14,15^ The phototherapy method is divided into photothermal therapy (PTT) and photodynamic therapy (PDT), among which PTT is more widely selected.^16,17^ PTT can use photothermal reagents to convert light energy into heat energy, so as to achieve local heating of tumors, and to use the poor heat tolerance of tumor cells to achieve the purpose of inhibiting tumor growth.^18,19^ The commonly used photothermal reagents include noble metal nanomaterials,^20,21^ carbon nanomaterials,^22,23^ transition metal sulfides,^24,25^ and organic dyes.^26,27^ Among them, transition metal sulfides represented as Cu_2_S are easy to prepare, and they have adjustable size and morphology, and high extinction coefficient and photothermal conversion efficiency in the near-infrared (NIR) region, which are ideal for the photothermal action.^28,29^

However, further applications of transition metal sulfides in PTT are still limited by the toxic surface ligand and poor colloidal stability.^30,31^ Surface modification and shell coating of NPs are widely used to solve these problems.^32–34^ For example, polyethylene glycol (PEG) and polyvinyl alcohol (PVA)-modified NPs have increased water solubility and stability.^35–37^ NPs can also be coated by a variety of shells, mainly into inorganic shells and polymer shells.^38–41^ SiO_2_ is the most common in the inorganic shells.^42^ The polymer shells mainly include polyaniline shells, polypyrrole shells and polydopamine shells.^43–45^ In addition to these, there are some amphiphilic polymers that can transfer oil phase NPs to the aqueous phase and achieve surface modification of NPs at the same time.^46–48^ Due to the toxicity of oil phase NPs, they cannot be directly used for diagnosis and treatment. This phase transfer process is conducive to improving the water stability and reducing the toxicity of NPs. After this treatment, the biocompatibility of NPs improves and the application of nanomaterials is expanded.^49,50^ Tween 20 (Tw20), a kind of biocompatible macromolecule, is one of the representative amphiphilic polymers and has been widely used.^51,52^

In this study, Cu_2_S@OLA NPs were prepared in an organic solvent under vacuum atmosphere. In addition, Cu_2_S@Tw20 NPs were successfully obtained by phase transfer of amphiphilic polymer Tw20. In comparison with Cu_2_S@OLA NPs, Cu_2_S@Tw20 NPs show improved water solubility and colloid stability. The modification of Tw20 also made it possible to load chemotherapeutic drugs,^53^ with the loading rate of doxorubicin (Dox) in Cu_2_S@Tw20 reaching 11.3%. The prepared Cu_2_S/Dox@Tw20 NPs have low toxicity to normal cells and can kill tumor cells under NIR light irradiation. Liver and kidney function tests further demonstrated the safety of nanocomposites. In *in vivo* tumor model experiments in nude mice, Cu_2_S/Dox@Tw20 NPs showed inhibitory effects on tumor growth due to loaded chemotherapy drugs, with an inhibition rate of 76.2%. Further, with combined NIR light, PTT and chemotherapy, the tumor was completely eliminated without recurrence. Histopathological analysis showed that the main organs of mice remained normal, without inflammatory cell infection and other phenomena, and our experiment was safe and reliable.

## 2 Experimental

### 2.1. Materials

Cu(i)Cl (99.9%) and Tween 20 were purchased from Aladdin. Oleylamine (OLA) (70%) and 1-octadecene (ODE) (90%) were purchased from Sigma-Aldrich. Sulfur powder (99%), hexane, ethanol and cyclohexane were purchased from Beijing Chemical Reagent Ltd. Hematoxylin & eosin dyeing solution was purchased from Nanjing Keygen. Cell culture medium, trypsin, cell counting kit-8 (CCK-8) and Annexin V-FITC/PI apoptosis assay kit were purchased from Beijing Solarbio Science & Technology Co., Ltd. Balb/c nude mice were purchased from Beijing Vital River Laboratory Animal Technology Company. Deionized water was used in all experiments.

### 2.2. Preparation of Cu_2_S@OLA NPs

First, 1 mmol Cu(i)Cl and 6 mL OLA were added into a three-necked flask called A flask. Then, 0.5 mmol sulfur powder and 2 mL ODE were added to another three-necked flask called B flask. Two flasks were connected to the vacuum line and after 10 min of deaeration, they were connected to the nitrogen line. This process was repeated 3 times to completely remove oxygen. The A and B flasks were then heated to 180 °C and 100 °C under nitrogen flow, respectively. After both CuCl and sulfur powder were completely dissolved, the heat was removed, allowing them to naturally cool to room temperature. The solution in the B flask was added to the A flask, and then the A flask was heated to 180 °C at a rate of 10 °C min^−1^ and continued for 15 min. Finally, the heat was removed, allowed to naturally cool to room temperature and the reaction was stopped. Ethanol was added to the solution and centrifuged (3500 g, 5 min). The supernatant was removed and the precipitate was dissolved in 5 mL hexane. This step needs to be repeated 2 times to thoroughly wash the product. The product ultimately needs to be dissolved in cyclohexane.

### 2.3. Preparation of Cu_2_S@Tw20 NPs

First, 40 mg Cu_2_S@OLA NPs were dissolved in 4 mL cyclohexane, and then 100 μL Tween-20 was added and sonicated for 10 min. This solution was added to 10 mL deionized water with vigorous stirring under ultrasound. After about 2 h in a 70 °C water bath, cyclohexane was completely evaporated, and the remaining solution was centrifuged at 14 000 g for 10 min, and the precipitate was dissolved in deionized water to store.

### 2.4. Preparation of Cu_2_S/Dox@Tw20 NPs

**Scheme 1:**
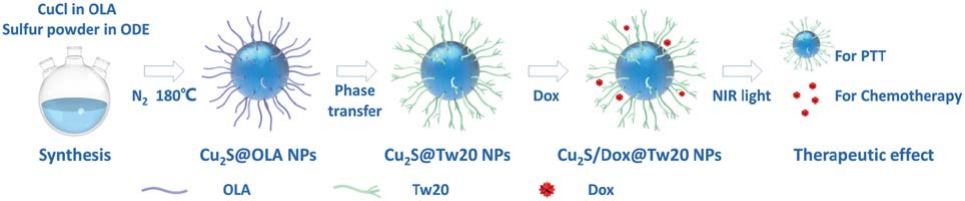
Schematic of the reactant, the preparation process of Cu_2_S@OLA NPs, Cu_2_S@Tw20 NPs, and Cu_2_S/Dox@Tw20 NPs, and the function mechanism in light-triggered photothermal therapy and chemotherapy.

First, 5 mL 2 mg mL^−1^ Cu_2_S@Tw20 NPs were stirred with a Dox solution of equal concentration and volume for 24 h. After centrifugation at 14 000 g for 10 min, the supernatant was removed, and the precipitate was re-dissolved in water for further use. Scheme 1 was used to show the structure of the nanoparticles.

### 2.5. *In vitro* cell experiments

In cytotoxicity tests, Ealy926 cells were mixed with Cu_2_S@Tw20 NPs and Cu_2_S/Dox@Tw20 NPs at different concentrations of 0, 25, 50, 100, 150, and 200 μg mL^−1^. After 24 h, the relative cell viability was determined by CCK-8 staining. Cytotoxicity was also evaluated by flow cytometry. Ealy926 cells were mixed with Cu_2_S/Dox@Tw20 NPs at different concentrations of 0, 100, and 200 μg mL^−1^. After 24 h, Ealy926 cells in each group were collected and washed by PBS. Then, 3 μL each of Annexin-V-FITC and PI were added into the cell suspension, respectively. After 15 min of culturing at room temperature, 400 μL annexin-binding buffer was added and the tests were carried out within an hour. In PTT *in vitro*, KB cells were cultured with Cu_2_S@Tw20 NPs and Cu_2_S/Dox@Tw20 NPs at 200 μg mL^−1^ for 30 min, and then irradiated by NIR laser for 5 min at different laser power densities of 0, 0.33, 0.5, 1, 2 and 3 W cm^−2^, respectively. After irradiation, the relative cell viability was also studied with CCK-8 staining. For flow cytometry, KB cells were mixed with Cu_2_S/Dox@Tw20 NPs at 200 μg mL^−1^ for 30 min, and then irradiated by NIR laser at 3 W cm^−2^ for different time periods of 0, 5, and 10 min. The procedure of cell collection and staining was consistent with the cytotoxicity test. All the tests were repeated three times.

### 2.6. Safety study *in vivo*

First, 250 μg of Cu_2_S@Tw20 NPs and Cu_2_S/Dox@Tw20 NPs were dispersed in 50 μL saline, respectively, and injected into the tail vein of the mice. After 24 h, 300 μL serum of the mice were separated for liver and kidney function tests. Each group had five samples and there are also five healthy mice of the same age as control.

### 2.7. *In vivo* pharmacokinetic study

A pharmacokinetic study was performed by measuring the amount of Cu_2_S/Dox@Tw20 NPs in blood. Simply the ratio between Cu_2_S/Dox@Tw20 NPs in the blood and the total amount in the injected solution was calculated, the Cu_2_S/Dox@Tw20 NP content in blood at different time points was studied, and then, blood circulation half-life time was calculated. Specifically, 50 μL 5 mg mL^−1^ of Cu_2_S/Dox@Tw20 NPs were intravenously injected into the mice. Then, 10 μL of blood was obtained from the tail vein of the mice at different time points of 0, 1, 2, 4, 8, 12, 18 and 24 h. These blood samples were dissolved in nitric acid to test the content of Cu(ii) by ICP-AES. After 24 h, the distribution of Cu_2_S/Dox@Tw20 NPs in KB tumors was also tested.

### 2.8. Animal experiments

A total of 15 Balb/c nude mice aged 4 weeks and weighing 18 g were purchased from Beijing Vital River Laboratory Animal Technology Company. All the animal procedures were performed in accordance with the Guidelines for the Care and Use of Laboratory Animals of Jilin University and approved by the Animal Ethics Committee of Jilin University. First, 1.5 million of KB cells were subcutaneously injected into the right hind leg of mice to establish an animal model. After 2 weeks of feeding, the tumor grew to 50 mm^3^, and the mice weighed about 18 g. The mice were randomly divided into 3 groups and named: control group, Cu_2_S/Dox@Tw20 NP group and Cu_2_S/Dox@Tw20 NPs + laser group, respectively. In the control group, 50 μL of normal saline was intravenously injected into the mice. As for Cu_2_S/Dox@Tw20 NP group and Cu_2_S/Dox@Tw20 NPs + laser group, 50 μL of 5 mg mL^−1^ Cu_2_S/Dox@Tw20 NPs were intravenously injected into the mice. After this, the tumors of the mice in the Cu_2_S/Dox@Tw20 NPs + laser group were irradiated with 808 nm laser for 30 min at a power density of 0.33 W cm^−2^. In the next 16 days, tumor volumes were measured every two days. After 16 days, all the mice were euthanized, and the tumors were dissected for H&E stain. It is worth mentioning that in the Cu_2_S/Dox@Tw20 NPs + laser group, one tumor was excised immediately after irradiation. Besides, the major organs of mice in every group were dissected for histopathological analysis to study the long-term bio-safety of Cu_2_S/Dox@Tw20 NPs.

### 2.9. Characterization

A JEOL JEM-2100F transmission electron microscope was used to perform transmission electron microscopic investigations. A Rigaku X-ray diffractometer was used to perform X-ray powder diffraction investigations. A Shimadzu 2600 UV-vis-NIR spectrophotometer was used to record UV absorption spectra. An 808 nm LEO diode laser was used to perform photothermal investigations. A Malvern Zetasizer NanoZS was used to perform zeta potential investigations. A PerkinElmer Optima 3300DV was used to perform ICP-AES investigations. A BD FACSCalibur was used to perform flow cytometry.

## 3 Results and discussion

### 3.1. Preparation and characterization of Cu_2_S/Dox@Tw20 NPs

**Fig. 1:**
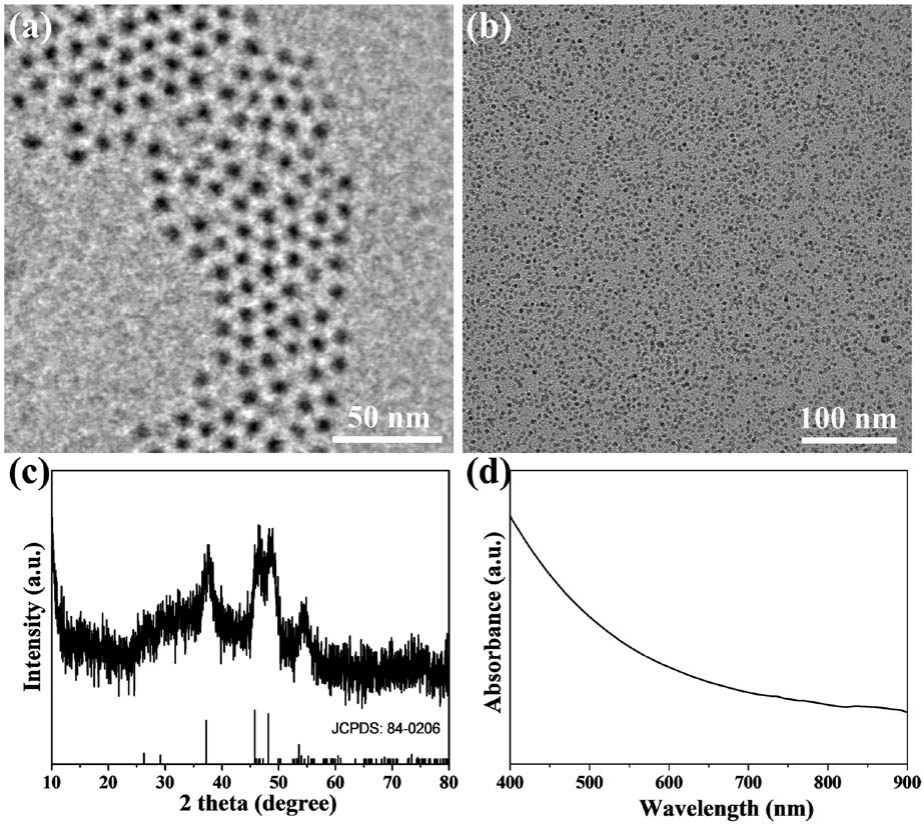
TEM image of Cu_2_S@OLA NPs (a) and Cu_2_S@Tw20 NPs (b). The scale bar is 50 nm. (c) XRD patterns of Cu_2_S@Tw20 NPs. (d) UV-vis-NIR absorption spectrum of Cu_2_S/Dox@Tw20 NPs.

In this study, Cu_2_S@OLA NPs were prepared in an organic solvent under vacuum. Transmission electron microscopy (TEM) showed that the prepared Cu_2_S@OLA NPs were uniform and the diameter was 5 nm (Fig. 1a). Cu_2_S@OLA NPs are insoluble in water, while Tw20 is an amphiphilic polymer. Cu_2_S@Tw20 NPs were obtained by a phase transfer method with Cu_2_S@OLA NPs and Tw20, and Cu_2_S@Tw20 NPs were soluble in water, which indirectly proves the successful coating of Tw20. After the phase transfer, the size and morphology of the NPs did not change significantly (Fig. 1b), and the average diameter was 5.02 nm (Fig. S1†). The hydration particle size of Cu_2_S@Tw20 NPs was 14.7 ± 1.3 nm and the PDI was 0.08. Because the hydration particle size is larger than 10 nm, nanoparticles will not be rapidly metabolized *in vitro* through kidney clearance, thus ensuring bioavailability and subsequent therapeutic effect, which is of great significance for biological application. The products were further tested using a X-ray powder diffractometer (XRD), and the results indicated that the XRD pattern was corresponding to Cu_2_S in the hexagonal crystal system (Fig. 1c). The prepared Cu_2_S@Tw20 NPs can be loaded with Dox. The drug loading efficiency was calculated by a standard curve method, and after many tests and calculations, it was calculated to be 11.3 ± 1.2%.

**Fig. 2:**
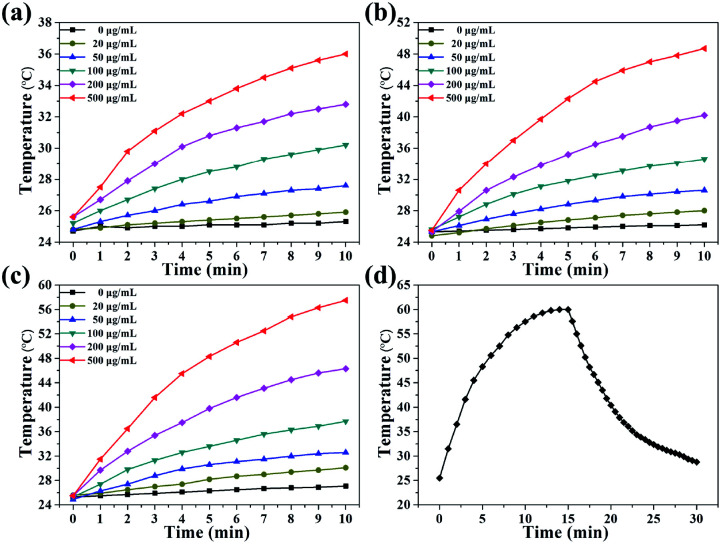
Temperature increment of Cu_2_S/Dox@Tw20 NPs by altering the concentration at a fixed power density of 1 W cm^−2^ (a), 2 W cm^−2^ (b), and 3 W cm^−2^ (c). (d) Photothermal conversion efficiency of Cu_2_S/Dox@Tw20 NPs is measured at a laser power density of 3 W cm^−2^ and a concentration of 500 μg mL^−1^. In the above experiments, all the temperatures were recorded at the interval of 1 min.

As revealed in Fig. 1d, Cu_2_S/Dox@Tw20 NPs have strong NIR absorption at around 808 nm, and also have high extinction capacity as well as photothermal conversion capacity. The concentration-dependent temperature increment was also exhibited to show the photothermal conversion capacity. As shown in Fig. 2a–c, the solution temperature increased with the increment in concentration at fixed laser power densities of 1, 2 and 3 W cm^−2^. This is because as the concentration of the solution increases, so does the collective heating effect. The horizontal comparison of the three figures showed that when the solution concentration is fixed, higher laser power can bring higher temperature rise, because the increase in laser power provides higher energy. The photothermal conversion efficiency of Cu_2_S/Dox@Tw20 NPs was further calculated. An electronic thermometer showed the temperature rise and fall of the Cu_2_S/Dox@Tw20 NP solution at a concentration of 500 μg mL^−1^ at 3 W cm^−2^ (Fig. 2d). Based on this, combining with the absorbance value of the solution, the photothermal conversion efficiency of Cu_2_S/Dox@Tw20 NPs was calculated to be 24.3% (Fig. S2†).

### 3.2. Cell experiments *in vitro*, bio-safety and pharmacokinetic study *in vivo*

**Fig. 3:**
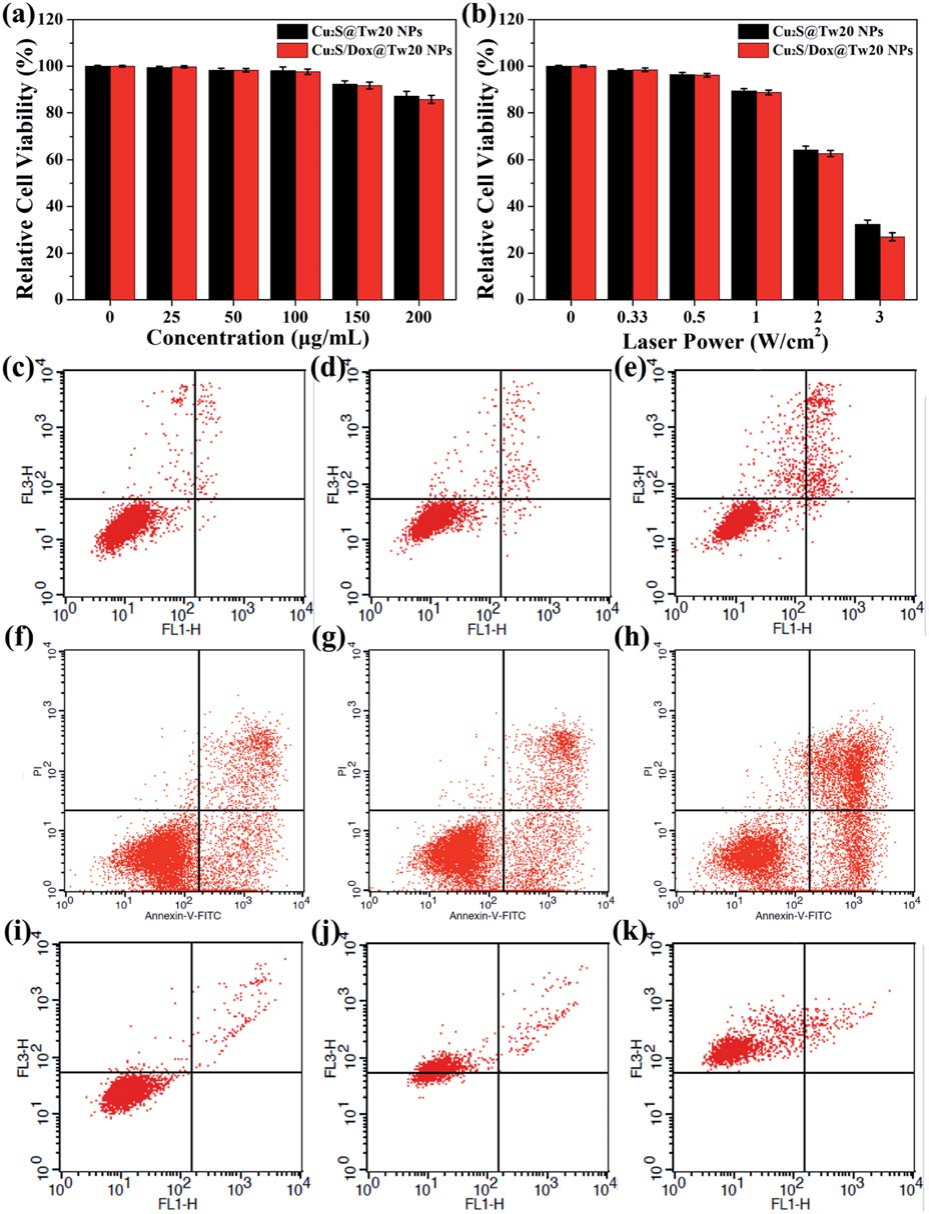
(a) Ealy926 cells were incubated with different concentrations of Cu_2_S@Tw20 NPs and Cu_2_S/Dox@Tw20 NPs for 24 h, and the relative cell viabilities were estimated by a CCK-8 assay. (b) KB cells were incubated with 200 μg mL^−1^ Cu_2_S@Tw20 NPs and Cu_2_S/Dox@Tw20 NPs for 30 min, respectively, and then irradiated by a 808 nm laser at different power densities for 5 min. The relative cell viabilities were estimated by a CCK-8 assay. (c–e) Ealy926 cells were mixed with Cu_2_S/Dox@Tw20 NPs at different concentrations of 0, 100, and 200 μg mL^−1^ for 24 h, and the cell viabilities were estimated by flow cytometry analysis. Percentages of living cells: (c) 97.94%, (d) 96.71%, and (e) 85.79%. (f–h) KB cells were mixed with Dox at different concentrations of 0.4, 0.8, and 1.6 μg mL^−1^ for 24 h, and the cell viabilities were estimated by flow cytometry analysis. The cell viabilities were estimated by flow cytometry analysis. The percentage of living cells: (f) 73.98%, (g) 64.58%, and (h) 44.83%. (i–k) KB cells were mixed with Cu_2_S/Dox@Tw20 NPs at a concentration of 200 μg mL^−1^ for 30 min, and then irradiated with NIR laser at a laser power density of 3 W cm^−2^ for different time periods of 0, 5, and 10 min. The cell viabilities are estimated by flow cytometry analysis. Percentage of living cells: (i) 95.48%, (j) 26.98%, and (k) 0%.

The cytotoxicity was first tested *in vitro* with human submesenteric artery endothelial cells (Ealy926). The cytotoxicity of Cu_2_S@Tw20 NPs and Cu_2_S/Dox@Tw20 NPs was estimated by a cell counting kit-8 (CCK-8) assay *via* culturing cells with NPs for 24 h at different concentrations. As shown in Fig. 3a, Cu_2_S@Tw20 NPs and Cu_2_S/Dox@Tw20 NPs had low cytotoxicity and the relative cell viability was still higher than 85% at 200 μg mL^−1^. The slightly higher toxicity of Cu_2_S/Dox@Tw20 NPs was due to the loaded chemotherapeutic drugs, which had a certain killing effect on normal cells. However, as can be seen from the figure, the increase in toxicity caused by chemotherapeutic drugs is minimal. This is because very little of the loaded Dox was released into the medium (Fig. S3†). Cytotoxicity was also evaluated by flow cytometry. When the concentrations of Cu_2_S/Dox@Tw20 NPs were respectively 0, 100 and 200 μg mL^−1^, the proportion of living cells gradually decreased to 97.94%, 96.71% and 85.79%, respectively (Fig. 3c–e). The therapeutic performance of Cu_2_S@Tw20 NPs and Cu_2_S/Dox@Tw20 NPs was tested *in vitro* with human oral epithelia carcinoma cells (KB). In this experiment, KB cells were irradiated with an 808 nm laser. As the increase in laser power, the relative cell viability decreased gradually after 5 min irradiation (Fig. 3b). The higher tumor cell killing effect of Cu_2_S/Dox@Tw20 NPs was due to the enhanced release of Dox under laser (Fig. S3†). As an efficient chemotherapeutic drug, Dox had a strong killing effect on KB cells. With the increase in Dox concentration, the relative cell viability gradually decreased (Fig. S4†), and the half maximal inhibitory concentration (IC_50_) was calculated to be 1.21 μg mL^−1^. After culturing with KB cells at different concentrations for 24 h, the flow cytometry also demonstrated the killing effect of Dox (Fig. 3f–h). With a fixed laser power density of 3 W cm^−2^ and a concentration of 200 μg mL^−1^, the cell viability decreased gradually with the increase in time. By flow cytometry, at the irradiation time of 0, 5 and 10 min, the proportions of living cells were 95.48%, 26.98% and 0%, respectively (Fig. 3i–k).

**Fig. 4:**
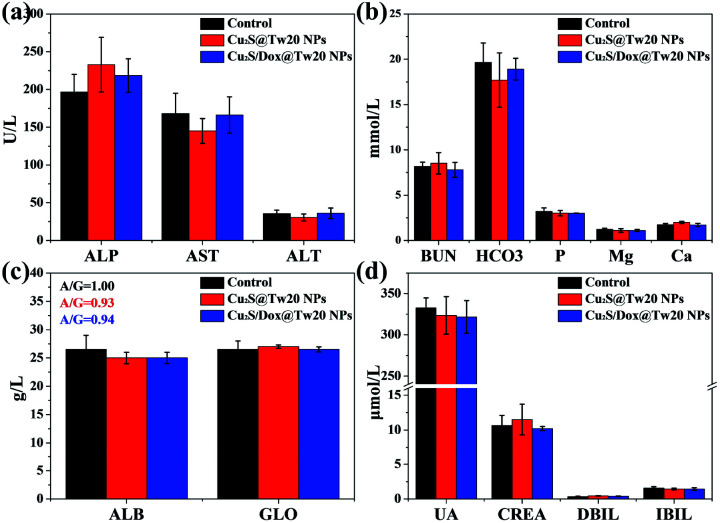
(a–d) Major liver and kidney functions indexes in liver and kidney functions tests. There were no significant differences among the indexes in each group. The control group is age-matched healthy mice.

The liver and kidney function tests can reflect short-term toxicity of the NPs. After 24 h of Cu_2_S@Tw20 NPs and Cu_2_S/Dox@Tw20 NPs injection intravenously into the mice, the serum was obtained for liver and kidney function tests. As revealed in Fig. 4a–d, the main liver and kidney function indexes such as albumin (ALB), globulin (GLO), uric acid (UA), creatinine (CREA), alkaline phosphatase (ALP), and alanine transferase (ALT) were all within the same level. There was no significant difference between the mice in the experimental groups and the control group, indicating that the short-term toxicities of Cu_2_S @Tw20 NPs and Cu_2_S/Dox@Tw20 NPs were low.

**Fig. 5:**
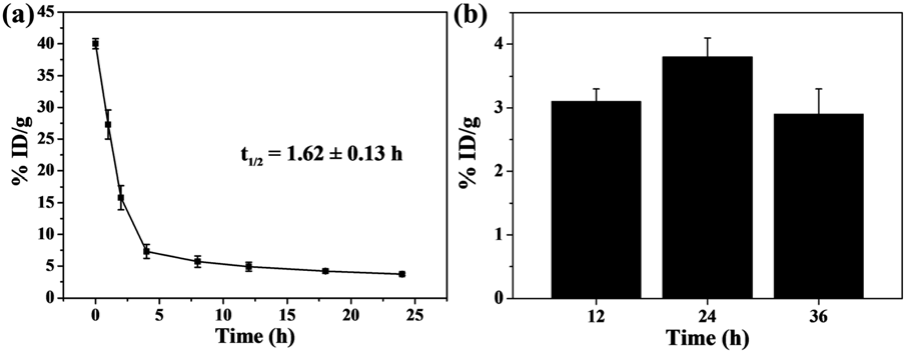
(a) Blood circulation of Cu_2_S/Dox@Tw20 NPs *in vivo*. (b) Biodistribution of Cu_2_S/Dox@Tw20 NPs in KB tumors 12, 24, and 36 h after injection. %ID per g: percent per gram of tissue.

The pharmacokinetic behavior of Cu_2_S/Dox@Tw20 NPs during the blood circulation was studied by monitoring the concentration of Cu_2_S/Dox@Tw20 NPs in the blood. The zeta potential shows that the potential of Cu_2_S/Dox@Tw20 NPs was −6.1 mV. NPs with negative surface can efficaciously avoid being marked and cleared in blood circulation.^15,54,55^ On the basis of inductively coupled plasma atomic emission spectroscopy (ICP-AES) results, the half-life of Cu_2_S/Dox@Tw20 NPs was calculated to be 1.62 ± 0.13 h, which was longer than most of the same class of NPs (Fig. 5a). Correspondingly, prolonged blood circulation time is in favor of the accumulation of nanomaterials in tumors by the EPR effect.^15,54,55^ In KB tumors, the retention rate showed an increasing trend and then a decreasing trend, and it was high at 24 h, which also indicated that laser should be applied after 24 h in PTT (Fig. 5b).

### 3.3. Animal experiments

**Fig. 6:**
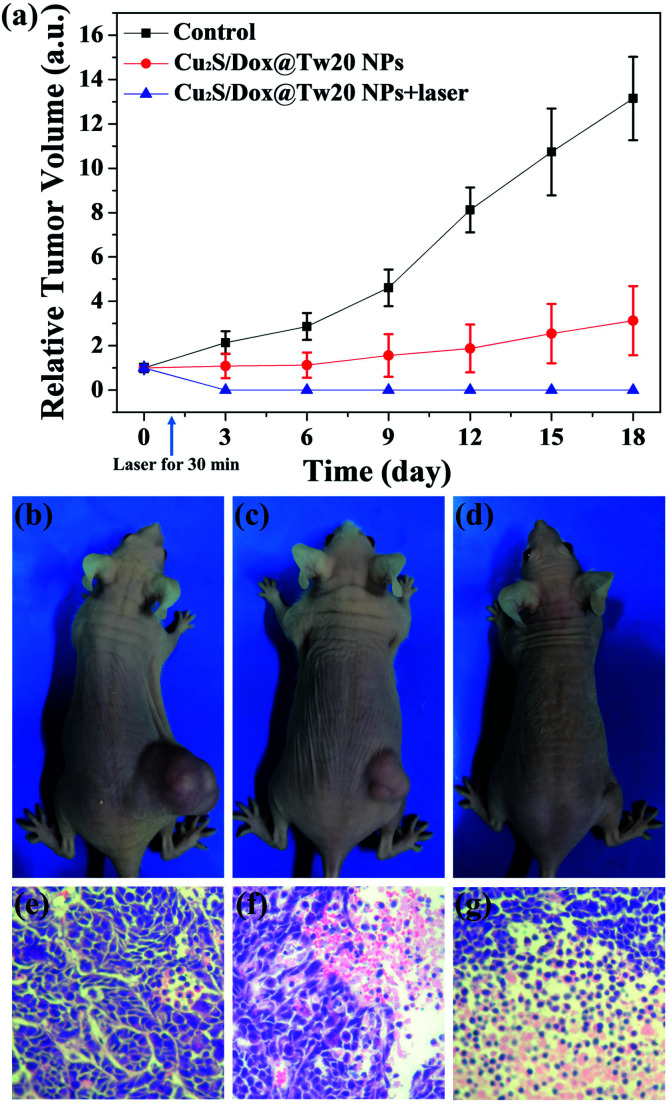
*In vivo* synergistic therapy. The mice were divided into 3 groups: control group, Cu_2_S/Dox@Tw20 NP group and Cu_2_S/Dox@Tw20 NPs + laser group. (a) Relative tumor volume growing curves of each group. (b–d) After 16 days, the photos of nude mice in each group. The average tumor volumes are 821, 203, and 0 mm^3^ for each group. In H&E-stained tumor slices, tumors are taken from the control and Cu_2_S/Dox@Tw20 NP groups in the 16th day after the injection (e and f). H&E-stained tumor slice for the Cu_2_S/Dox@Tw20 NPs + laser group performed immediately after laser irradiation (g). Magnification: 200 times.

**Fig. 7:**
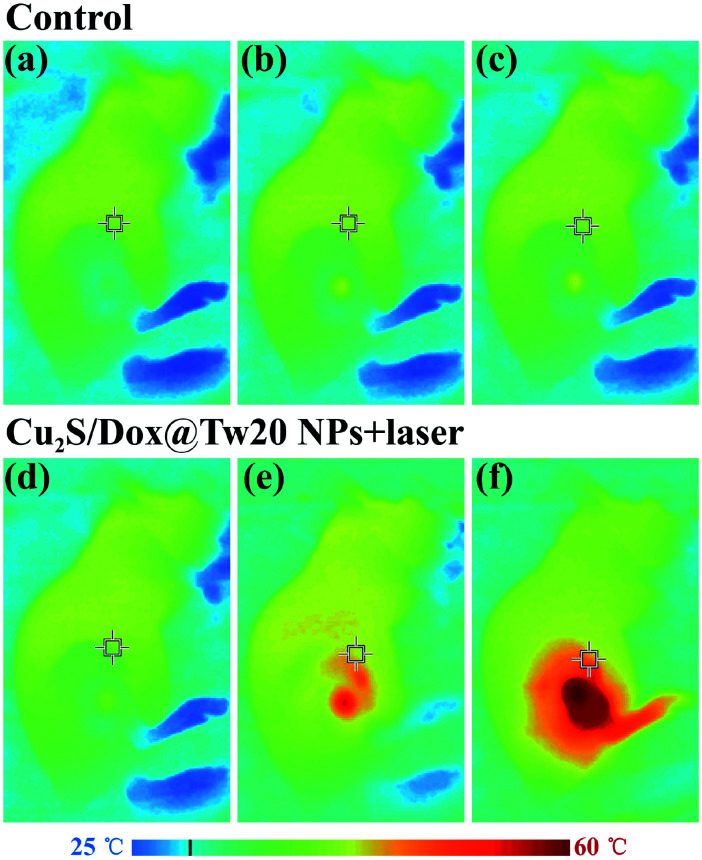
Infrared thermal images of the mice treated with saline and 250 μg Cu_2_S/Dox@Tw20 NPs in 50 μL saline under the irradiation of 808 nm laser at a power density of 0.33 W cm^−2^ for 0 min (a and d), 15 min (b and e) and 30 min (c and f).

*In vivo* tumor therapy experiments were performed on nude mice. In the control group, mice were treated with only normal saline. The tumor growth curve within 16 days is shown in Fig. 6a. The tumors in the control group grew very quickly, and after 16 days, the average volume of tumors reached 821 mm^3^ (Fig. 6a and b). The hematoxylin–eosin (H&E)-stained tumor section showed that all tumor cells were in the normal state (Fig. 6e). Compared with the control group, tumor growth trend of the mice injected with Cu_2_S/Dox@Tw20 NPs was slower, and the tumor growth was significantly inhibited. After 16 days, the average tumor size was 203 mm^3^ (Fig. 6a and c). This is because the negatively charged surfaces facilitate the accumulation of Cu_2_S/Dox@Tw20 NPs in tumor sites. Cu_2_S/Dox@Tw20 NPs in the tumors can release the loaded Dox slowly and inhibit the tumor growth by chemotherapy. The H&E-stained tumor section also shows partial apoptosis of tumor cells (Fig. 6f). However, chemotherapy alone is not enough to completely eliminate the tumor. Therefore, the NIR laser was introduced for the combination of photothermal therapy and chemotherapy. In the control group, the tumor heating rate was quite slow; even after 30 min of irradiation, the tumor temperature was still less than 40 °C (Fig. 7a). However, after 15 min of irradiation under 0.33 W cm^−2^, tumors in the Cu_2_S/Dox@Tw20 NPs + laser group showed a fast temperature increment over 40 °C. Under the continued laser irradiation for further 15 min, the tumor temperature reached 60 °C and extensive necrosis of tumor cells were shown in the tumor section stained with H&E (Fig. 7b and 6g). The chemotherapeutic drugs were released in response to the heat and acted on the tumor site for several days. The postoperative chemotherapy effectively reduced tumor recurrence. There was no recurrence within 1 month, which indicates that Cu_2_S/Dox@Tw20 NPs were ideal photothermal reagents.

**Fig. 8:**
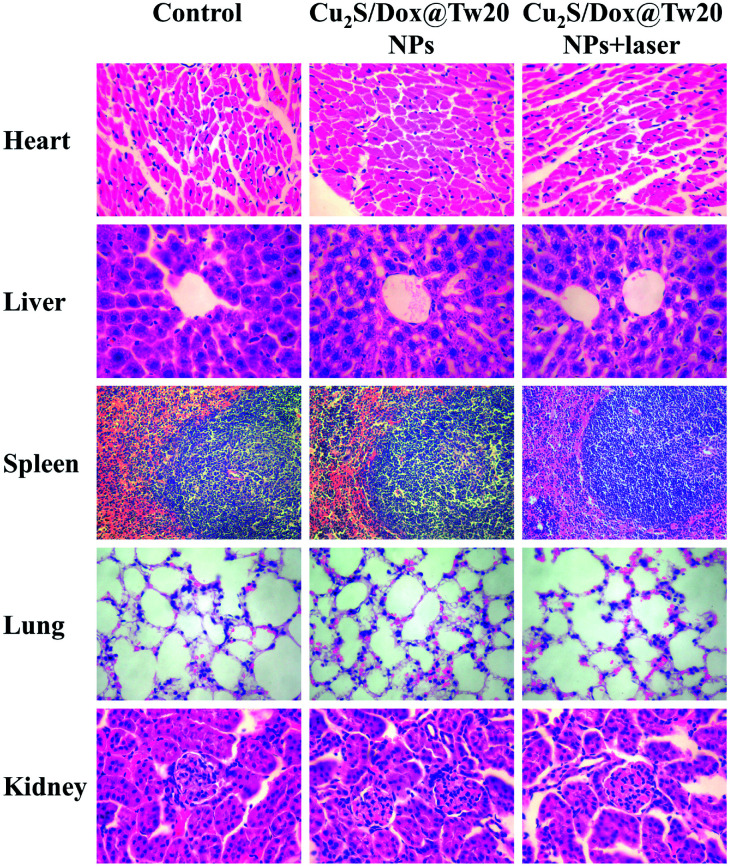
H&E-stained splanchnic slices after 16 days. The control group is age-matched healthy mice. Magnification: 400 times for the heart, liver, lung, and kidney, and 200 times for the spleen.

The biosafety of Cu_2_S/Dox@Tw20 NPs was also performed by H&E-stained organ slices. There was no inflammatory cell infiltration observed in major organs like the heart, liver, spleen, lungs and kidneys. Although the liver cells have some steatosis, it is not caused by the toxicity of the nanomaterials. In the lung samples, there are red blood cells in the interstitial vessels of the lung, not inflammatory cells. Compared with healthy mice, all the cells in these organs were at the same status (Fig. 8). This confirms that Cu_2_S/Dox@Tw20 NPs are of good safety in tumor PTT.

## 4 Conclusion

In summary, we demonstrated the fabrication of Cu_2_S/Dox@Tw20 NPs with excellent tumor therapeutic functions. The modification of Tw20 not only improves the biocompatibility and physiological stability, but also makes it possible to load the chemotherapeutic drug Dox, the drug loading rate of which is 11.3%. The prepared Cu_2_S/Dox@Tw20 NPs have low toxicity to normal cells and can kill tumor cells under NIR light irradiation. Liver and kidney function tests further demonstrated the bio-safety. The negative charge on the surface of Cu_2_S/Dox@Tw20 NPs prolongs the half-life of blood circulation, which is beneficial for the enrichment of NPs in tumors. In animal experiments, Cu_2_S/Dox@Tw20 NPs showed inhibitory effects on tumor growth due to loaded chemotherapy drugs, with an inhibition rate of 76.2%. Further combined with NIR light, the tumor was completely eliminated with the synergistic effect of PTT and chemotherapy. Histopathological analysis shows no hydroncus or inflammatory cell infiltration and our experiment is bio-safe.

## Author contributions

B. S. and Y. L. proposed and supervised the project. S. W. L. and L. W contribute equally to this paper. They designed and performed the experiments and co-wrote the paper. B. Z., Z. W. and Y. Y. W. participated in most experiments. All authors have given approval to the final version of the manuscript.

## Conflicts of interest

There are no conflicts of interest to declare.

## Supplementary Material

RA-010-D0RA03069D-s001
