# Supplementary material for: Doxorubicin-loaded Cu2S/Tween-20 nanocomposites for light-triggered tumor photothermal therapy and chemotherapy
Source: RSC Adv. 2020 Jul 10;10(44):26059–66. doi: 10.1039/d0ra03069d (PMC9055350; doi:10.1039/d0ra03069d)
Supplement: RA-010-D0RA03069D-s001 [file RA-010-D0RA03069D-s001.pdf]

Electronic Supplementary Material (ESI) for RSC Advances.
This journal is © The Royal Society of Chemistry 2020

Supporting Information

Doxorubicin Loaded Cu_2S/Tween-20 Nanocomposites for Light Triggered Tumor Photothermal Therapy and Chemotherapy

Shuwei Liu,[c,#] Lu Wang,[b,#] Bin Zhao,[b] Ze Wang,[c] Yinyu Wang,[d] Bin Sun[a,b,*] and Yi Liu[c]

[a]Department of Oral and Maxillofacial Surgery, School and Hospital of Stomatology, Jilin University, Changchun, 130021, P. R. China, [b]Jilin Provincial Key Laboratory of Tooth Development and Bone Remodeling, Jilin University, Changchun, 130021, P. R. China, [c]State Key Laboratory of Supramolecular Structure and Materials, College of Chemistry, Jilin University, Changchun 130012, P. R. China, [d]School of Stomatology, Baicheng Medical College, Baicheng 137000, P. R. China.

*Address correspondence to sunbin06@sohu.com.

[#]These authors contribute equally.

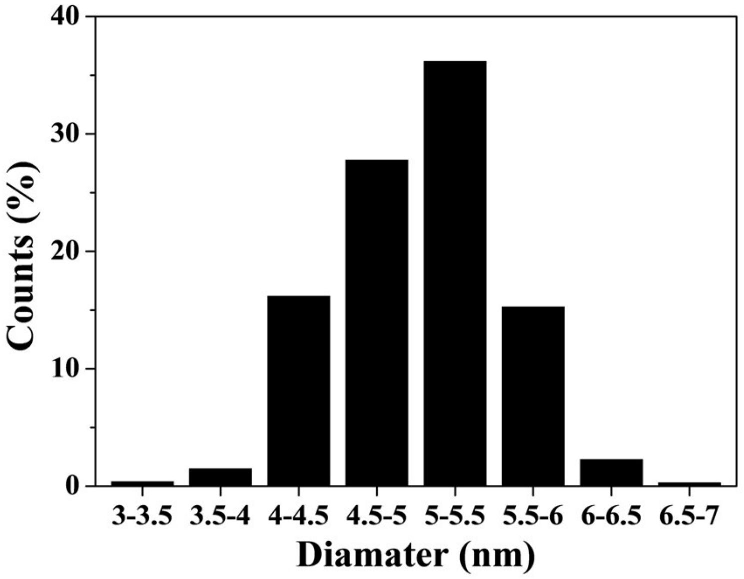

Figure S1. The size distribution of Cu_2S/Dox@Tw20 NPs measured from TEM photos.

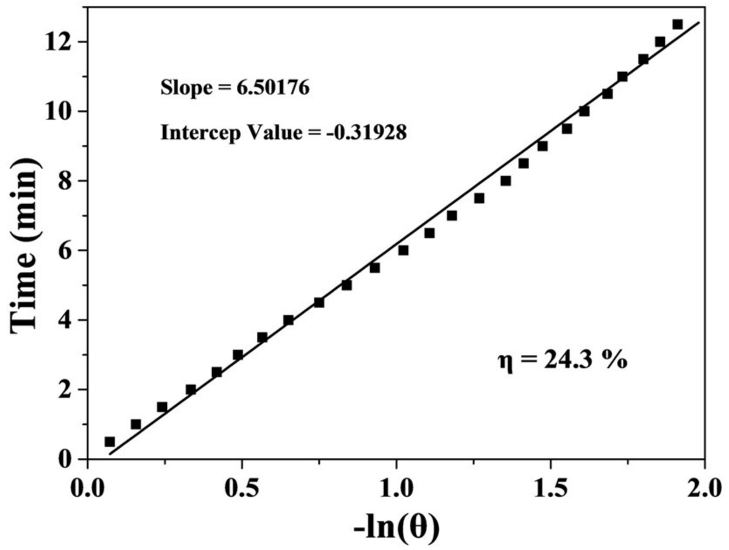

Figure S2. Time constant for heat transfer of Cu_2S/Dox@Tw20 NPs is determined by applying the linear time data from the cooling period versus negative natural logarithm of driving force temperature. Based on this, the photothermal conversion efficiency was calculated as 24.3 %.

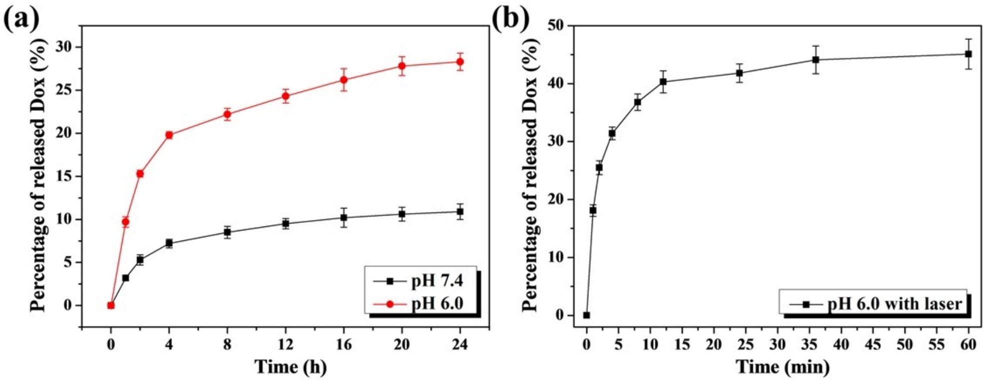

Figure S3. (a) Drug release curves of Dox under different pH environment. (b) Drug release curve of Dox under laser treatment at the pH of 6.0.

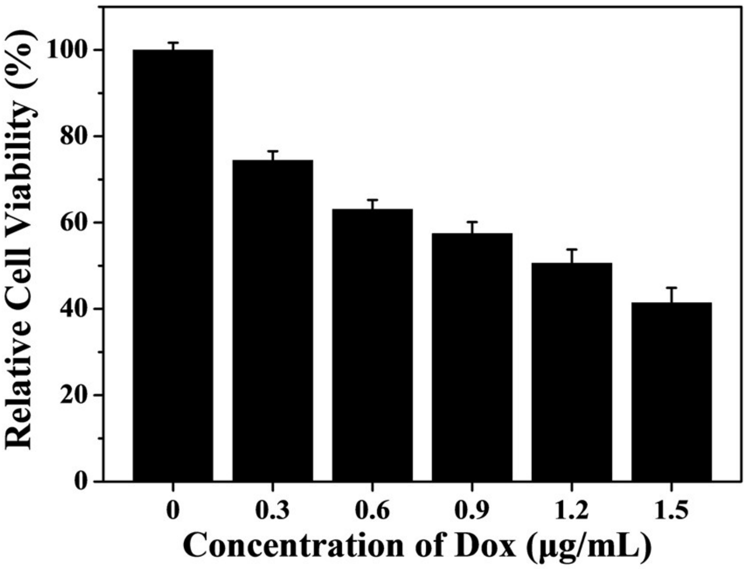

Figure S4. KB cells were mixed with Dox at different concentrations of 0, 0.3, 0.6, 0.9, 1.2 and 1.5 μg/mL for 24 min, and then the relative cell viabilities are estimated through CCK-8 assay.
